# Dissolving porcine and human microthrombi by short exposure to microdoses of alteplase in an in vitro model of microvascular obstruction

**DOI:** 10.1038/s41598-025-03060-1

**Published:** 2025-05-24

**Authors:** Anastasia Milusev, Yannick Rösch, Yves Kuster, Petra Wolint, Jens Ulmer, Miriam Weisskopf, Nikola Cesarovic, Dominik Obrist

**Affiliations:** 1 ARTORG Center for Biomedical Engineering Research, University of Bern, Freiburgstrasse 3, 3008 Bern, Switzerland; 2 Institute for Microtechnology and Photonics, OST University of Applied Sciences, Buchs SG, Switzerland; 3 Department of Health Sciences and Technology, Swiss Federal Institute of Technology, Zurich, Switzerland; 4 Center for Surgical Research, University Hospital Zurich, University of Zurich, Zurich, Switzerland; 5 Department of Cardiothoracic and Vascular Surgery, German Heart Center Berlin, Berlin, Germany

**Keywords:** Microcirculation, Microfluidics, Alteplase, Intracoronary, In vitro, Thrombolysis, Myocardial infarction, Drug delivery

## Abstract

Coronary microvascular obstruction (MVO) occurs in up to 57% of patients suffering from ST-segment elevation myocardial infarction (STEMI). One cause for MVO is distal embolization by microthrombi after percutaneous coronary intervention (PCI) of the infarct-related coronary artery. MVO is associated with an adverse cardiac prognosis post-STEMI. However, there are no evidence-based therapies for MVO, presenting an unmet therapeutic need. We investigated a novel pharmacotherapeutic approach to resolve embolizing MVO using an in vitro microfluidic model with porcine or human microthrombi to investigate thrombolysis with alteplase, a fibrinolytic drug. We show that a brief (90 s) exposure to concentrated microdoses of alteplase significantly reduces microthrombus size by up to 75%. 50% lysis occurred within six to twelve minutes after alteplase exposure depending on the initial microdose. Our results suggest that delivering a therapeutic drug directly to the microvasculature to briefly achieve high local drug concentrations has the potential to address embolizing MVO. The combination of short drug exposure and high local concentration is possible with a recently developed infusion system based on intracoronary controlled flow infusion (CoFI). CoFI uses microdoses of alteplase that are up to 1300 times lower than intravenous doses and could thereby minimize bleeding risk.

**Supplementary Information:**

The online version contains supplementary material available at 10.1038/s41598-025-03060-1.

## Introduction

**Fig. 1:**
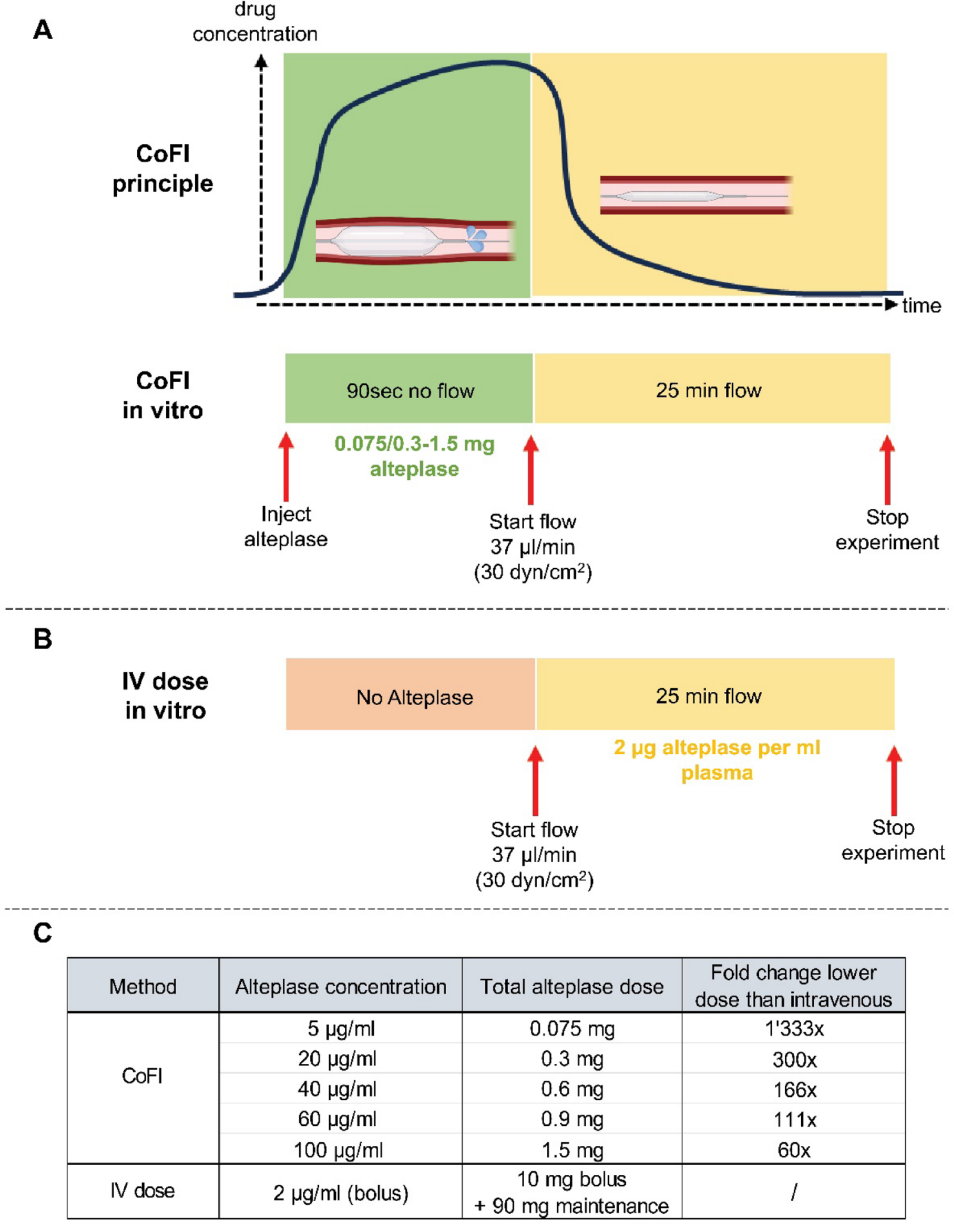
Experimental set-up and dosing protocol for thrombolysis under flow. (**A**) Representation of the CoFI principle (top) and experimental protocol for mimicking CoFI in the microfluidic chip (bottom) with different alteplase microdoses. (**B**) Protocol for mimicking intravenous (IV) drug administration by perfusion with citrated plasma containing 2 µg/ml alteplase (IV dose). (**C**) Overview of alteplase concentrations and microdoses and a calculation (right column) showing the amount of dose reduction for CoFI compared to an IV dose. The total alteplase dose is calculated from the concentration multiplied by 15 ml representing the clinical CoFI infusion volume (i.e. 5 µg/ml * 15 ml = 0.075 mg total dose). For IV administration, a bolus dose (10 mg) is administered followed by a maintenance dose (90 mg) = total dose of 100 mg, however the highest drug concentration in the bloodstream is reached by the bolus dose (10 mg in an average blood volume of 5 L = 2 µg/ml).

Cardiac microvascular obstruction (MVO) of the coronary vasculature occurs in 57% of patients treated for ST Segment Elevation Myocardial Infarction (STEMI) by percutaneous coronary intervention (PCI)^1^. MVO has several underlying causes, one being obstruction due to embolizing microthrombi (MT)^2^. MVO leads to under-perfusion of the myocardial tissue, thereby exacerbating damage to the heart after myocardial infarction (MI)^3^. Although it is known that MVO correlates with negative clinical outcomes in STEMI, diagnosis and treatment of MVO is challenging. Currently, the gold standard for diagnosis of MVO is late-gadolinium-enhanced cardiac magnetic resonance imaging (cMRI)^4^ which is not performed immediately after MI, making timely treatment of MVO impossible. Moreover, only limited treatment options^5^ with potentially harmful side effects^6,7^ exist. As embolizing MVO affects small vessels (less than 300 µm^8–10^), mechanical intervention to remove clots causing the obstruction is not feasible. Therefore, a possible therapeutic approach is the use of thrombolytic agents. One example is recombinant tissue-type plasminogen activator (r-tPA), which is only active when bound to fibrin. Once bound, tPA converts plasminogen to plasmin which degrades fibrin to soluble fibrin degradation products on the clot surface leading to thrombolysis^11^. Alteplase^12^ and Tenecteplase^13^ are r-tPA which are indicated for MI and have been in use for decades, however, with limited success^14,15^. Fibrinolytic drugs in MI are administered as an intravenous (IV) bolus followed by an IV maintenance infusion^16^. While they decrease mortality after MI, systemic thrombolysis was recognized as a risk for systemic bleeding^17^. As a result, the cardiovascular field sought other therapeutic options and routes of administration. This led to trials with intracoronary (IC) drug infusion aiming to deliver smaller and localized doses of fibrinolytic drugs while decreasing systemic side effects. Multiple clinical trials have been conducted investigating catheter-directed IC infusion of tPA for the treatment of MVO. However, these trials showed mixed results and little benefit in reduction of MVO which was rarely measured as the primary outcome^18–22^. One potential issue is that even with IC infusion the drug does not reach obstructed microvessels. Schmitz-Rode et al.^23^ have shown that a drug bolus is washed out of the proximity of obstructed vessels within 0.5 s before it can induce thrombolysis. It is likely that the same effect occurs in IC drug injection for MVO therapy. Recently, a new catheter-based approach for IC drug infusion using a novel multi-lumen balloon catheter was developed by CorFlow Therapeutics AG, Baar, Switzerland. The purpose of this approach is to deliver fluids directly to the coronary vasculature by controlled flow infusion (CoFI) and delay drug washout. CoFI works by occluding the vessel lumen with a balloon upstream of the MVO employing a multi-lumen catheter. During the occlusion, a drug is infused via the multi-lumen catheter distally of the balloon occlusion (Fig. 1A, top). The occlusion is maintained for no longer than 90 s, which assures that no ischemic damage is introduced^24^. During this occlusion, high local drug concentrations are maintained in the coronary vessels. Importantly, by directly infusing drugs intracoronary, CoFI uses drug microdoses which represent a fraction of IV administered total drug doses, achieving a drastic drug reduction (Fig. 1C). In vitro, use of the CoFI catheter was shown to increase local infusate concentrations in MVO affected microvessels by 58% and prevent immediate washout while maintaining high concentrations for more than a minute^25–27^. This in vitro study was conducted with a passive dye, validation of CoFI with pharmacologically active drugs for thrombolysis is still outstanding. It is currently unknown whether the short 90 s contact with concentrated drugs which can be achieved by CoFI is enough to induce thrombolysis and how fast after the treatment first effects occur.

The motivation for our in vitro experiments was to translate the novel clinical methodology of CoFI into a controllable and observable in vitro environment to investigate thrombolysis. For this, we have used a microfluidic model that closely recapitulates the real size and fluid dynamics of the coronary microcirculation. MT, one of the main causes of MVO, derived from porcine or human blood were treated inside the microfluidic model with concentrated microdoses of the thrombolytic drug alteplase to mimic CoFI (Fig. 1A, bottom). The results from this in vitro study will inform future clinical trials using CoFI to treat MVO in patients with STEMI.

## Results

### Porosity and composition of in vitro produced clots

**Fig. 2:**
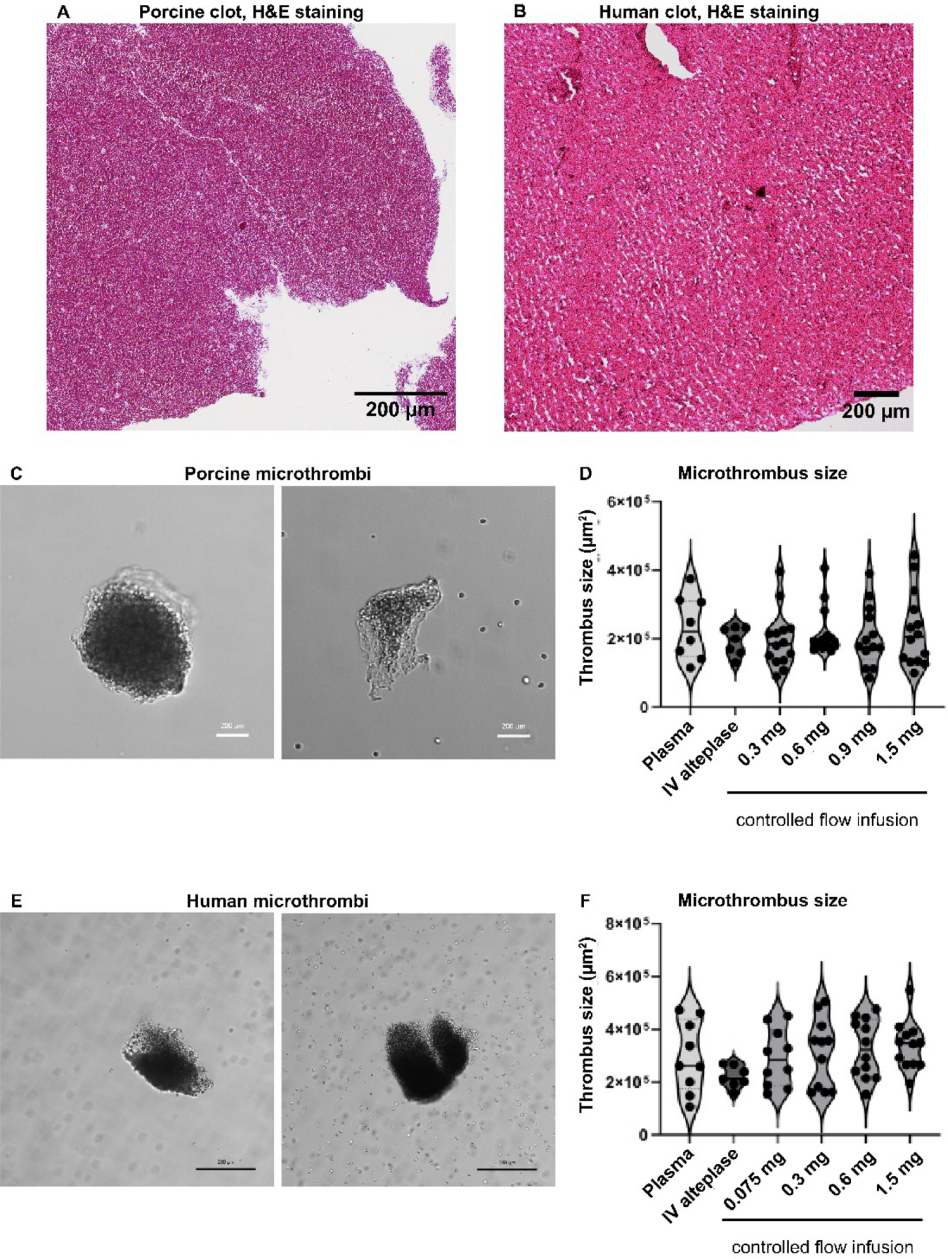
Characterization of porcine and human microthrombi (MT) used for thrombolysis experiments. (**A**, **B**) Representative images of H&E stained (**A**) porcine and (**B**) human clots. (**C**, **E**) Representative light microscopy images of freshly produced (**C**) porcine and (**E**) human MT. (**D**, **F**) Size in µm^2^ of (**B**) porcine and (**D**) human microthrombi used for microfluidic experiments was measured from experimental videos using Fiji. Data are pooled from CoFI and combined approach experiments representing eight to fourteen independent experiments with five to six different blood donors. Kruskal–Wallis test with multiple comparisons was used for statistical analysis. Scale bar: 200 µm.

An important factor that influences susceptibility to thrombolysis is clot retraction/density and clot composition^28,29^. Hematoxylin and Eosin (H&E) staining of our in vitro created porcine and human thrombi shows high density of erythrocytes (red), fibrin (pink) is visible around erythrocyte aggregates and some immune cells are present in the clot (Fig. 2A,B for porcine and human clots respectively). This composition is comparable to similarly created in vitro clots described by others^28–30^. The macroscopic clots used to create MT for our in vitro experiments were created in Eppendorf tubes, Sutton et al.^28^ have shown that surface properties influence the degree of clot retraction. To evaluate clot retraction/porosity, the percentage of white area within H&E stained macroscopic clots was quantified. Both porcine and human clots consisted of 11–14% white area. This corresponds to unretracted or mildly retracted blood clots as reported by Sutton et al.^28^ or Mercado-Shekhar et al.^29^.

As embolizing MVO occurs in the microvasculature with vessel diameters below 100 to 300 µm^9,10^, we worked with MT of about 200–300 µm in size. To analyse MT size and variability, the projected area of MT inside the microfluidic chip was quantified. While the size of individual MT was variable (Fig. 2C,E), the average projected area in µm^2^ was comparable for the different experimental conditions (Fig. 2D,F).

### Alteplase microdoses lead to lysis of porcine and human microthrombi under flow

To investigate CoFI in vitro, porcine and human MT were treated with different microdoses of alteplase for 90 s and perfused with porcine or human plasma (Fig. 1A,C). To mimic clinical IV administration of alteplase (IV dose) another set of experiments was performed where MT were perfused with porcine or human plasma containing 2 µg/ml alteplase without prior treatment with alteplase (Fig. 1B,C). We also tested CoFI in addition to perfusion with plasma containing 2 µg/ml alteplase, mimicking CoFI combined with the administration of an IV bolus dose (CoFI + IV dose). The total CoFI alteplase microdose is calculated based on a 15 ml CoFI infusion volume (for example 20 µg/ml * 15 ml = 0.3 mg total dose). This microdose is administered only once at the beginning of the experiment and corresponds to a CoFI treatment as it would be performed in a STEMI patient.

**Fig. 3:**
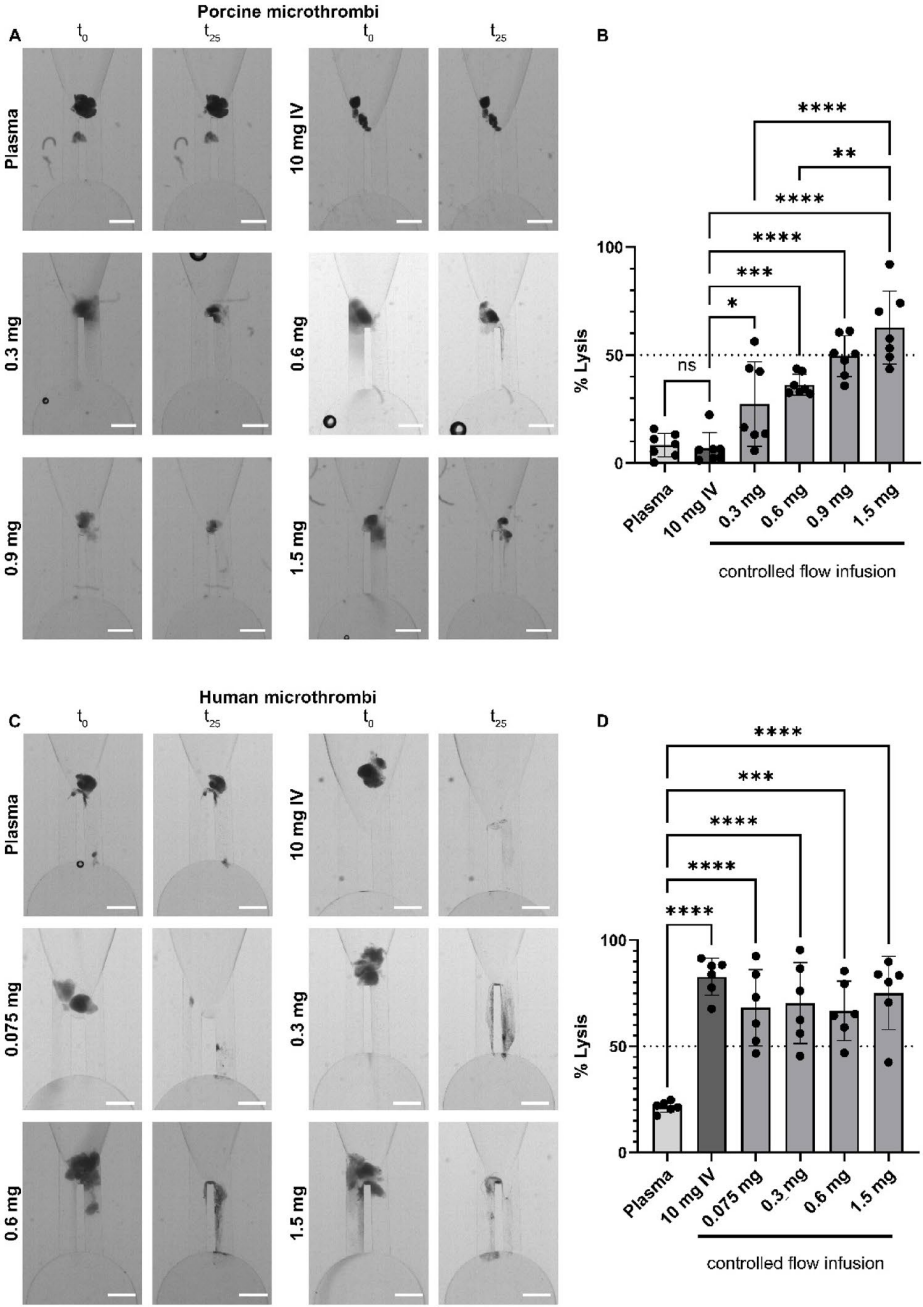
Thrombolysis of porcine and human MT under flow treated with controlled flow infusion. MT were incubated for 90 s with various alteplase microdoses and subsequently perfused with porcine (**A**, **B**) or human (**C**, **D**) citrate plasma. (**A**, **C**) Representative images of MT inside the microfluidic channel before (t_0_) and after (t_25_) 25 min perfusion. (**B**, **D**) Quantification of MT lysis after 25 min. 10 mg IV (dark grey bar) was not incubated with alteplase but perfused with citrate plasma containing 2 µg/ml alteplase. The control (Plasma) was not incubated with alteplase and perfused with plasma alone. Data are from six to seven independent experiments and six different blood donors. One-way ANOVA with multiple comparisons was used for statistical analysis. **** *p* < 0.0001, *** *p* < 0.001, ** *p* < 0.01, * *p* < 0.05. Scale bar 500 µm.

Figure 3A,C shows images of clots before (t_0_) and after (t_25_) incubation with alteplase microdoses representing CoFI and subsequent plasma perfusion. Components of the MT washed away during perfusion leading to a decreased MT area for all alteplase microdoses. MT stayed intact for controls (Plasma). Quantification of lysis shows that CoFI causes significantly higher thrombolysis than the IV dose (10 mg IV) for porcine (Fig. 3B) but not for human MT (Fig. 3D). The IV dose for porcine MT did not show more thrombolysis than the control (Fig. 3B; Plasma) which was not treated with alteplase. In contrast, for human MT, a similar amount of lysis is achieved with CoFI and with an IV dose (Fig. 3D, mean lysis 82%). This shows that exposing human MT but not porcine MT to a prolonged low concentration of alteplase causes equivalent thrombolysis to a short drug incubation (CoFI, 90 s). Additionally, mean human MT lysis of 68–75% was observed for all alteplase microdoses, indicating no dose dependent increase in lysis within the tested range. In contrast, thrombolysis increased for porcine MT with increasing CoFI alteplase microdoses. The highest alteplase microdose (1.5 mg) achieved an average of 63% lysis, which was significantly higher than the two lowest alteplase microdoses (27% and 36% for 0.3 mg and 0.6 mg, respectively).

Comparing human with porcine MT shows that with CoFI, human MT are significantly more susceptible to thrombolysis than porcine MT for alteplase microdoses of 0.3 mg and 0.6 mg (Fig. 3B,D). For the highest tested alteplase microdose (1.5 mg) there is no difference between human and porcine MT. To understand if there was any influence of the initial MT size on lysis efficacy, MT size in relation to MT lysis was evaluated (Suppl. Fig. S2A–E for porcine MT and S2F-J for human MT). Linear regression analysis and Spearman correlation index show no significant influence of MT size on lysis efficacy. Only the 0.6 mg microdose for human MT showed a weak correlation between lysis efficiency and initial MT size.

**Fig. 4:**
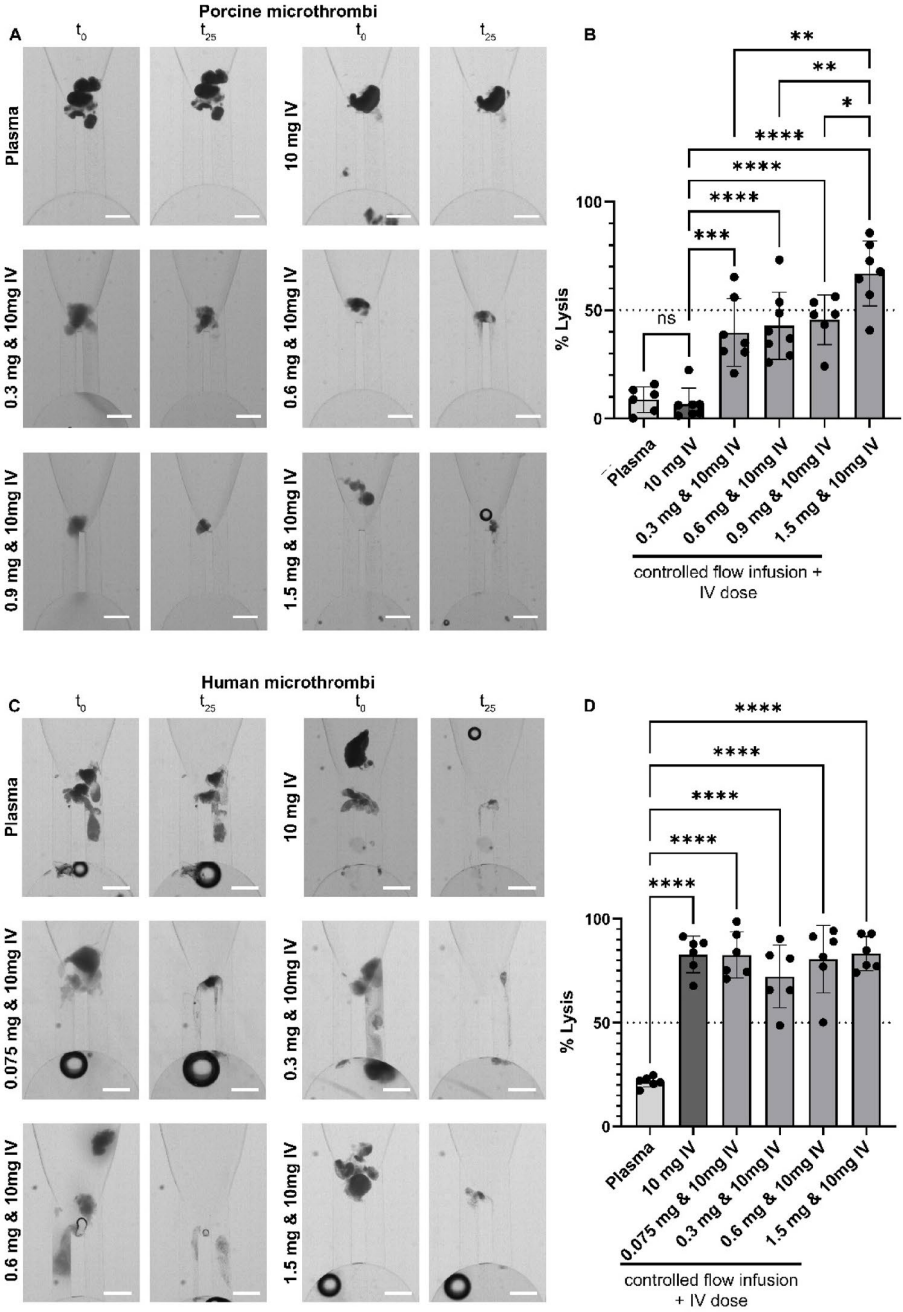
Thrombolysis of porcine and human MT under flow treated with a combined approach (CoFI + IV dose). MT were incubated for 90 s with various alteplase microdoses and subsequently perfused with porcine (**A**, **B**) or human (**C**, **D**) citrate plasma containing 2 µg/ml alteplase. (**A**, **C**) Representative images of MT inside the microfluidic channel before (t_0_) and after (t_25_) 25 min perfusion. (**B**, **D**) Quantification of MT lysis after 25 min. One control condition (Plasma) was not incubated with alteplase and perfused with porcine plasma alone. Data are from six to eight independent experiments and five different blood donors. One-way ANOVA with multiple comparisons was used for statistical analysis. ****p < 0.0001, ***p < 0.001, **p < 0.01, *p < 0.05. Scale bar 500 µm.

Next, we investigated the thrombolytic efficacy of CoFI combined with an IV dose (Fig. 4). Adding an IV dose to CoFI (CoFI + IV dose) increases the total alteplase dose 60- to 1333-fold (Fig. 1C), potentially impacting lysis efficiency. Images of the MT before and after alteplase treatment and plasma perfusion (Fig. 4A,C) show reduction in MT size for all alteplase microdoses but not for untreated MT (Plasma). CoFI + IV dose demonstrated significantly higher thrombolysis than the IV dose alone for porcine (Fig. 4B) but not for human MT (Fig. 4D). In contrast to previous results (Fig. 3), thrombolysis with the combined approach was only partially dose dependent for porcine MT. The three lowest alteplase microdoses (0.3–0.9 mg) induced comparable thrombolysis with an average of 36–43%. The highest alteplase microdose (1.5 mg) caused the strongest lysis with an average of 67%, which was significantly higher than the lysis achieved with all other alteplase microdoses. Thrombolysis of human MT was not dose dependent with a combined approach (Fig. 4D, mean MT lysis of 72–83%). Overall, there is no significant increase in thrombolysis of the combined approach compared to CoFI alone after 25 min of perfusion for both porcine and human MT (Suppl. Fig. S3). Again, human MT are significantly more susceptible to thrombolysis than porcine MT (Fig. 4) for all except the highest alteplase microdose.

In summary, these results show that CoFI using a single microdose of alteplase achieves 60–80% thrombolysis of porcine and human MT.

### Combined CoFI + IV approach influences lysis dynamics over time more strongly for porcine than human microthrombi

**Fig. 5:**
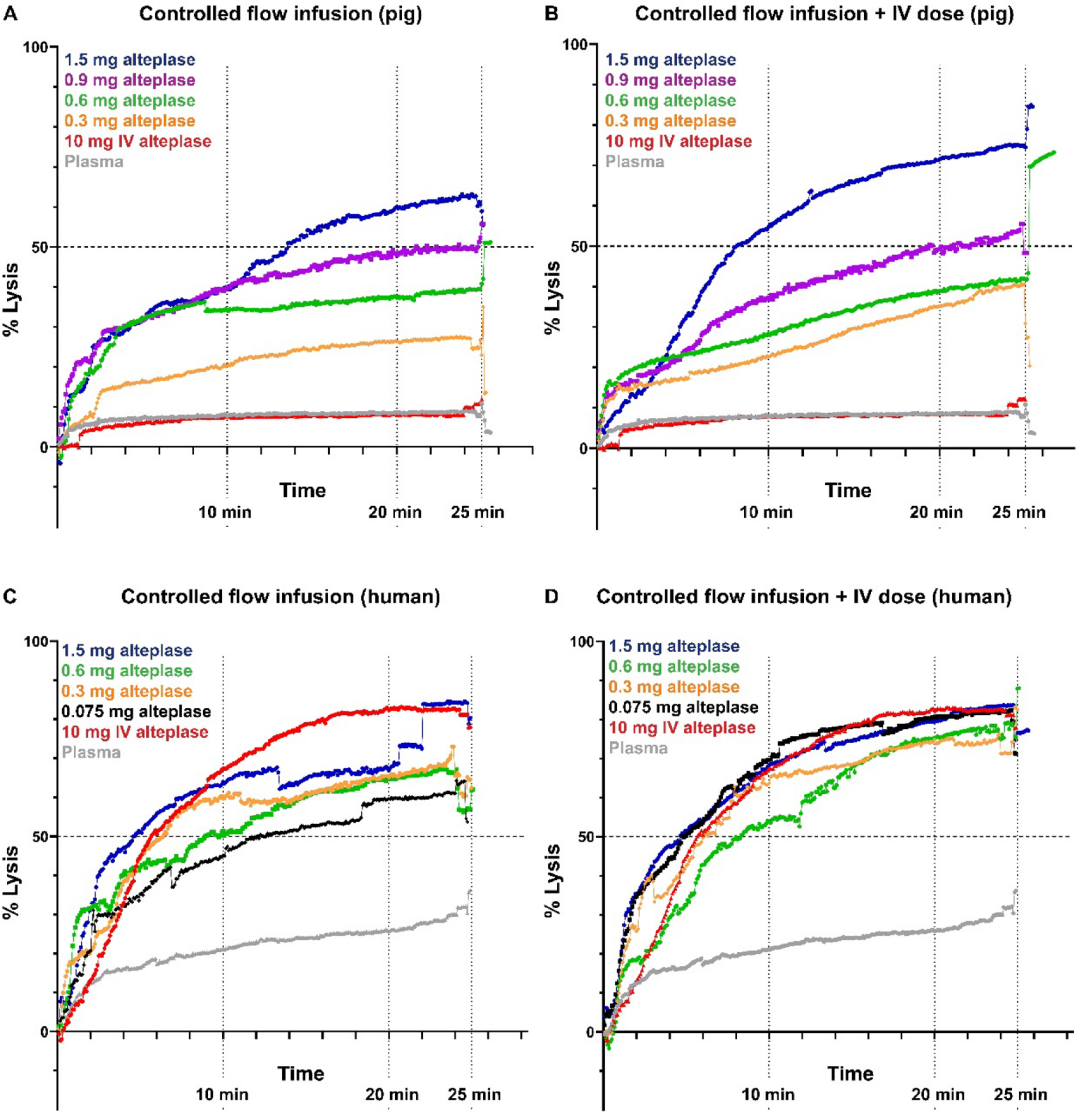
Thrombolysis of MT over time comparing controlled flow infusion to a combined approach (CoFI + IV dose). Thrombolysis over time for (**A**, **B**) porcine or (**C**, **D**) human MT treated with different alteplase microdoses and perfused with (**A**, **C**) plasma or (**B**, **D**) plasma containing 2 µg/ml alteplase. Each curve represents the mean lysis over time of at least 6 independent experiments. Curves including the standard deviation are shown in the Supplementary Figure S4.

To understand the dynamics of thrombolysis we measured MT lysis over time. For porcine MT, the CoFI (Fig. 5A) and the CoFI + IV dose (Fig. 5B) show distinct lysis over time curves for all the different alteplase microdoses. For human MT, this effect is much weaker (Fig. 5C,D) and strong lysis occurs within the first 10 min. Interestingly, for porcine MT, 50% thrombolysis is reached faster with the combined approach (8 min, Fig. 5B, blue curve) compared to CoFI alone (12 min, Fig. 5A, blue curve). In contrast, the difference is smaller for human MT where a combined approach only accelerates lysis for the lowest alteplase microdose (Fig. 5D, black curve). For human MT, the highest alteplase microdose in the combined approach (Fig. 5C,D blue curve) achieved 50% thrombolysis already after 4 min. This is 2 times faster than for porcine MT in the combined approach (Fig. 5B) and 3 times faster than for CoFI alone (Fig. 5A). Supplementary Figure S4 shows all lysis over time curves including standard deviations.

Overall, the combined approach leads to significantly faster overall thrombolysis than CoFI alone for all alteplase microdoses except for 0.6 mg (Supplementary Table S1).

### Local high alteplase concentrations are tolerated by endothelial cells

**Fig. 6:**
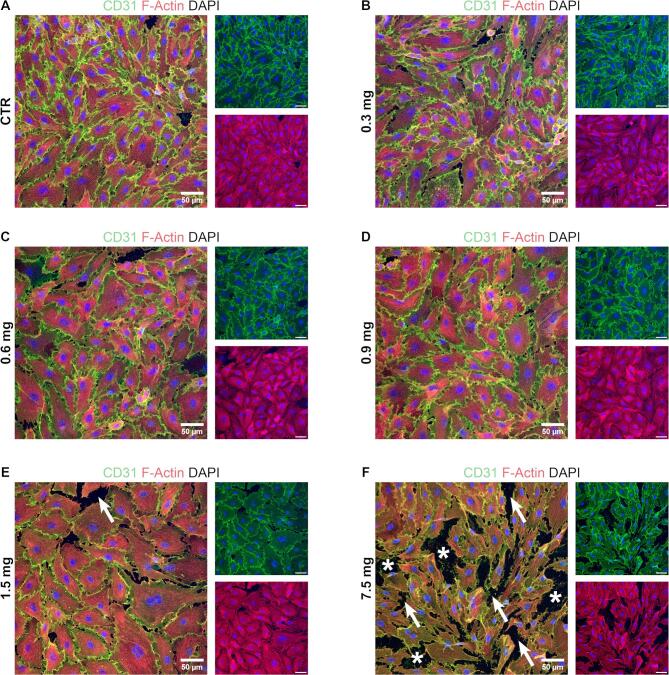
Staining of endothelial junctions on porcine arterial endothelial cells (PAECs) after incubation with various alteplase microdoses. (**A**–**F**) Representative images of PAECs grown in chamber slides until confluent, treated for 10 min with alteplase microdoses ranging from 0.3 to 7.5 mg and stained by immunofluorescence. CD31 is stained in green, F-actin in red and nuclei are stained in blue (DAPI). Arrows indicate disrupted cell junctions and asterisks indicate areas where cells detached. Images were acquired with a Zeiss LSM980 confocal microscope at 20 × magnification. Scale bar 50 µm.

**Fig. 7:**
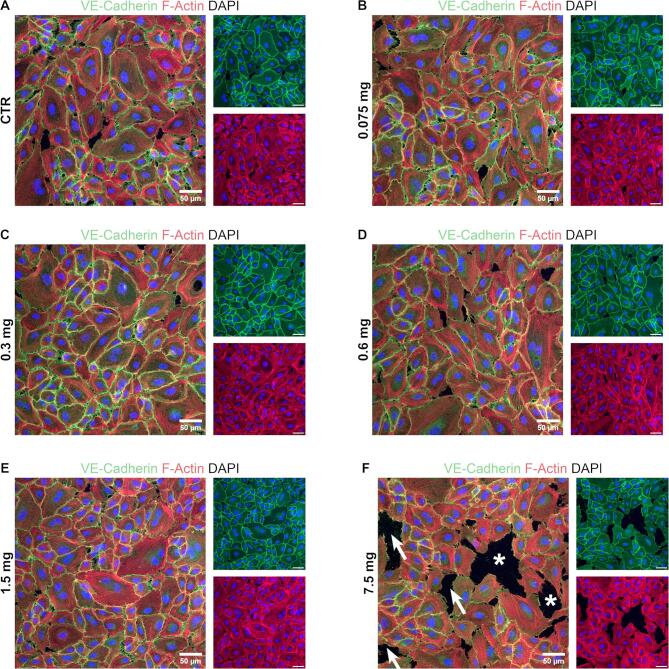
Staining of endothelial junctions on human arterial endothelial cells (HAECs) after incubation with various alteplase microdoses. (**A**–**F**) Representative images of HAECs grown in chamber slides until confluent, treated for 10 min with alteplase microdoses ranging from 0.075 to 7.5 mg and stained by immunofluorescence. VE-Cadherin is stained in green, F-actin in red and nuclei are stained in blue (DAPI). Arrows indicate disrupted cell junctions and asterisks indicate areas where cells detached. Images were acquired with a Zeiss LSM980 confocal microscope at 20 × magnification. Scale bar 50 µm.

A concern of using high local drug concentrations is the impact on the vascular environment. Our highest alteplase concentration is fifty times higher than the highest blood concentration in a clinical setting, raising the question of local tolerance. The first contact site is the vessel wall including endothelial cells (ECs). Yoeruek et. al^31^ showed that human corneal ECs in vitro were damaged by concentrations of alteplase above 125 µg/ml. To determine the tolerance of both porcine arterial ECs (PAECs) (Fig. 6) and human arterial ECs (HAECs) (Fig. 7) for alteplase in vitro, ECs were incubated with drug concentrations corresponding to CoFI alteplase microdoses used in MT lysis experiments. EC junctions showed intact and uniform staining for all microdoses up to 1.5 mg (corresponding to 100 µg/ml alteplase concentration) for PAECs (Fig. 6A–E) and HAECs (Fig. 7A–E). ECs incubated with five times higher microdoses (7.5 mg, equivalent to 500 µg/ml alteplase concentration) display patchy junction staining (white arrows, Figs. 6F and 7F) and gaps between cells indicating cell detachment (white asterisks, Figs. 6F and 7F). Our results show that alteplase microdoses of up to 1.5 mg (corresponding to concentrations of up to 100 µg/ml) are tolerated by both porcine and human arterial ECs, whereas higher doses lead to disruption of EC junctions and cell detachment.

## Discussion

In this work, we demonstrate that microthrombi, an important contributor to the development of MVO after myocardial infarction, are susceptible to lysis by alteplase at high local concentrations and very brief treatment times.

The novelty of this study is that thrombolysis is possible with a very short exposure to high alteplase concentrations. The combination of short exposure and locally high concentration is feasible with a very small drug bolus (microdose) administered by the CoFI system. These results have an impact on the understanding of how drug therapy for embolizing MVO can be administered most efficiently. We show that long drug exposure (as achieved by IV infusion) is not necessary for alteplase to exert its function. Instead, delivery of a therapeutic drug directly to the MT for only 90 s at high local drug concentrations causes thrombolysis and has the potential to address MVO caused by embolizing MT. Clot lysis in vitro has so far been performed over longer incubation times of 10 min and with much higher drug concentrations^32,33^. Our data suggests that activation of the thrombolytic process can occur very fast or that drug activity can be maintained even after washout of alteplase. Indeed, it was shown that plasminogen localizes within thrombi, bound to both fibrin and platelets and that upon initiation of thrombolysis with tPA there is accumulation of plasminogen^34^. Together with shear force due to plasma flow, this leads to destruction of the thrombus^34,35^. Furthermore, tPA bound to fibrin is protected against its natural inhibitor plasminogen activator inhibitor 1 (PAI1) present in plasma, indicating that tPA within the thrombus remains active over time even in the presence of inhibitors^35^.

An important factor that influences rt-PA lysis efficacy is clot retraction, with unretracted clots being more porous and susceptible to thrombolysis than retracted clots^28,29^. Clot retraction has been observed in vitro^36^ and described for in vivo coronary blood clots^37^ where retraction increased with time after clotting as shown by increasing occurrence of compressed red blood cells in the shape of polyhedrocytes, a sign of clot retraction. Clots that were up to 2 h old showed almost no retraction while clots older than 12 h were highly retracted^38^.

We show that our in vitro created clots are unretracted or mildly retracted (Fig. 2), suggesting that they are readily susceptible to thrombolysis and likely resemble fresh arterial thrombi which are not yet retracted. This is relevant in the context of STEMI where treatment is usually initiated within 6 h of symptom onset, suggesting that these clots are rather fresh and possibly not highly retracted. However, it is difficult to investigate the exact amount of clot retraction in vivo, making a direct comparison of retraction for in vitro and in vivo clots difficult.

Another factor which influences lysis efficacy is clot composition, but it is very challenging to compare in vitro to in vivo clot composition because in vivo clots are highly heterogeneous^39^. Moreover, very little data on the composition of MT exists. One study^40^ showed that embolized coronary thrombi consist of platelets and fibrin, but this study did not quantify erythrocytes. Although our in vitro created clots do not replicate the in vivo composition precisely, they contain major components like fibrin (the target of tPA) and erythrocytes.

In addition to clot retraction and composition, we have observed important differences in the susceptibility to thrombolytic treatment of porcine- compared to human MT for all tested alteplase microdoses (Figs. 3 and 4). Although the human and porcine cardiovascular system share similarities^41^, pig blood was shown to be hypercoagulable compared to human blood, with increased fibrinogen levels but low fibrinolysis^42,43^ and delayed activation of plasminogen^44^. In accordance with this, we observed 1.2 to 2.5 times higher thrombolysis for human MT compared to porcine MT. This is in line with research from others showing that human clots experience sixfold higher clot mass loss than porcine clots upon treatment with alteplase^43^. Neto-Neves et al.^45^ showed that porcine clots necessitate higher alteplase concentrations to achieve comparable thrombolysis to human clots. Likewise, we have seen that only the highest tested alteplase microdose (1.5 mg) caused comparable thrombolysis for human and porcine MT (Figs. 3 and 4). For all lower microdoses, human MT lysis was significantly stronger than porcine MT lysis. Furthermore, porcine MT also showed dose-dependent increase in lysis (Figs. 3 and 4), confirming that alteplase dose influences lysis efficiency. In contrast, human MT demonstrated consistently high lysis for all tested alteplase microdoses (Figs. 3 and 4), suggesting a saturation in the thrombolytic potential for human MT within the tested range. It would be interesting to test microdoses below 0.075 mg (lowest tested microdose) to understand the limit for human MT lysis.

All these results support a CoFI approach for thrombolysis of MT, but we have also observed that treatment of human (but not porcine MT) equivalent to an IV dose was sufficient to induce thrombolysis (Fig. 4). This suggests that a low drug concentration (2 µg/ml) over a longer time (25 min) can achieve thrombolysis as efficiently as CoFI for human MT but not for porcine MT. In line with our results, Huang et al.^43^ have shown that human thrombi can be lysed by perfusion with human plasma containing 3.15 µg/ml alteplase. Porcine clots perfused with the same alteplase concentration in porcine plasma did not exhibit thrombolysis. Yet, clinical data has thus far failed to show a meaningful impact of IV or IC therapy on MVO as detected by cMRI. In fact, in an IV or IC clinical setting, in contrast to most in vitro models, circulating blood containing alteplase reaches MT less efficiently due to impaired perfusion. Therefore, CoFI is crucial to deliver fibrinolytic drugs to MT in the MVO area and maintain high concentrations while preventing drug washout for long enough to initiate thrombolysis.

To achieve relevant systemic drug concentration clinically, high IV bolus doses of alteplase including a maintenance dose must be administered. In contrast, CoFI can be achieved with extremely low total alteplase microdoses that are more than 1000-fold lower than an IV dose.

To investigate a possible benefit of CoFI treatment added to standard IV treatment on MT lysis, we performed lysis experiments combining CoFI with an IV dose (2 µg/ml alteplase in plasma) (Fig. 4). A combined approach accelerates MT lysis dynamics without influencing the total amount of lysis (Fig. 5 and Suppl. Fig. S3), suggesting it can open up blocked microvessels up to 1.5 times earlier, thereby reducing ischemic time. However, sudden reperfusion of blocked vessels can reduce myocardial recovery and increase arrythmias^46,47^, suggesting that accelerating thrombolysis is not necessarily beneficial. In addition, a combined approach increases the total alteplase dose up to 1′333-fold for human and 300-fold for porcine MT (for the lowest tested microdose) compared to CoFI alone. This potentially increases systemic bleeding risk, without increasing the absolute amount of thrombolysis. Therefore, such an approach for the treatment of embolizing MVO should be evaluated critically.

One concern with the CoFI approach is the local use of concentrated alteplase, which could potentially lead to extravasation of erythrocytes across already damaged microvasculature and cause intramyocardial hemorrhage (IMH). The extravascular accumulation of erythrocytes in IMH causes a prolonged inflammatory reaction due to iron degradation products and occurs in up to 50% of STEMI patients after PCI^48^. IMH in addition to MVO has recently been recognized as an important contributor to MACE and mortality^49^. Although some studies show a correlation between the use of platelet inhibitors and IMH^50,51^, limited data exists for alteplase in a coronary context. McCartney et al.^52^ observed that patients receiving a large (20 mg) intracoronary dose of alteplase show an increase in IMH compared to placebo. But this was only observed for a subgroup treated late (> 4 h) after symptom onset. In fact, Basso et al.^53^ propose that ischemic time and reperfusion in general rather than thrombolytic use is associated with IMH. Importantly, both the alteplase dose and concentration in the study of McCartney et al. are more than ten times higher than what was used in our in vitro experiments. Additionally, we show that high local alteplase concentrations do not damage EC junctions (Figs. 6 and 7), suggesting that CoFI with alteplase would not aggravate any pre-existing EC damage and is therefore not expected to increase erythrocyte extravasation. Nonetheless, a contribution of alteplase to the development of IMH cannot be excluded and should be investigated in future studies.

Our study has several limitations: First, we worked exclusively with an in vitro model. Although our model does not recreate all physiological in vivo conditions adequately, it does represent the physiological fluid dynamics and size scale of the coronary microvasculature affected by embolizing MVO. Importantly, it allows insight into thrombolysis under flow in controlled conditions and isolated from confounders that might occur in vivo. Moreover, the present in vitro model was not created to replace in vivo animal studies. It was rather designed to establish a quantitative baseline for in vivo studies and provide relevant information which drug concentrations (in the given therapeutic setting with short incubation time) may be able to achieve significant thrombolysis. These results will help to design effective experimental protocols for animal trials and interpret in vivo data.

Second, we created blood clots in vitro. We have shown that our in vitro clot model is comparable to certain clots reported in literature, but shear stress is lacking in our method of creating clots. This is likely to have an impact on clot composition and retraction which influences susceptibility to thrombolysis.

Third, we have used citrated plasma instead of whole blood for perfusion. Plasma is established as a biological fluid and has been used for numerous in vitro microfluidic thrombolysis studies^32,33,54^. However, whole blood contains additional cells and proteins which can interfere with thrombolysis. For example, platelets can contribute to a pro-thrombotic effect after alteplase treatment due to activation of platelets and thrombin by alteplase^55,56^. This was proposed by McCartney et al.^19^ as a potential cause for the results obtained in their T-TIME study which investigated IC alteplase administration. We have observed attenuation of mean human clot lysis by about 10% in vitro in presence of not pre-activated platelets (data not shown). This indicates that there is an inhibitory effect of platelets on MT lysis. Nonetheless, there is still significant thrombolysis suggesting that similar results can be expected in vivo in the blood circulation. Finally, the research presented here focused on the use of a fibrinolytic drug. The microfluidic system can be used as a platform to test other drug classes such as platelet inhibitors. Importantly, a thrombolytic approach to treat MVO is viable for MVO caused by embolizing or in situ formed MT. However, MVO has also other causes which are not addressed by the therapeutic approach presented in this article. Therapy for other causes for MVO such as ischemia/reperfusion injury or edema^2^ necessitates further research and distinct treatment.

In conclusion, our in vitro model shows that significant thrombolysis of MT is achieved with short but highly concentrated alteplase treatment. Therefore, a CoFI approach would be ideal to deliver thrombolytic drugs to MT in embolizing MVO. Further and ongoing studies focus on the treatment of MVO caused by MT in a pig model based on alteplase microdoses established with the research described in this article. These results will guide CoFI clinical trials^57^ aiming to evaluate therapy for embolizing MVO in patients treated for STEMI.

## Material and methods

### Microfluidic chip design

A microfluidic chip was fabricated by micro milling from Polymethylmethacrylate (PMMA) substrates (top acryl, Schönenwerd, Switzerland) with outer dimensions of 100 × 1 mm. The channel structure milled into the PMMA surface was covered with a 1 mm PMMA substrate (top acryl, Schönenwerd, Switzerland) using a 3-APTES PDMS bonding process. This method yielded a rectangular channel covered by a flat PMMA plane. Due to the milling process, the surface roughness was on the order of micrometres. The finished channel consists of a straight inlet with a width of 1 mm and a height of 700 µm. The inlet leads into a chamber of tapered height ranging from 700 µm down to 30 µm followed by two outflow channels (300 µm wide by 30 µm high) assuring constant perfusion through the microfluidic channel (Suppl. Fig. S1). Injected MT (typical size of 200–300 µm) get stuck in the tapered area of the chip and remain non-occlusive permitting continuous flow around the MT throughout the experiment.

### Microthrombus production in vitro

MT were produced from citrated porcine or human whole blood similarly to a method described previously^58^, but with in vitro created blood clots. Porcine citrated whole blood was obtained from non-terminal experiments performed by a research group at the University of Zurich approved by the local Committee for Animal Experimental Research (Cantonal Veterinary Office Zurich, Switzerland) and in accordance with the University of Zurich guidelines and Swiss federal regulations. Pigs were purchased from a local commercial farm. Pigs were sedated with an intramuscular injection of Ketamine (15 mg/kg BW), Azaperon (2 mg/kg BW) and Atropine 1% (0.05 mg/kg BW). Prior to orotracheal intubation, anaesthesia was induced with a bolus of Propofol (1–2 mg/kg BW). Anaesthesia was then maintained with Isoflurane (1–2 Vol%) and a continuous infusion of Propofol (3 mg/kg BW). Buprenorphine 0.01 mg/ kg BW was administered intravenously for perioperative analgesia. The study is reported in accordance with ARRIVE guidelines. Human citrated blood was purchased anonymised from the Swiss Red Cross (SRK, Bern, Switzerland) where it was taken by licensed personnel in accordance with relevant guidelines and handled in accordance with Swiss federal regulations and University of Bern guidelines. According to Swiss legislation (Federal Office of Public Health) use of anonymised human biological material for non-genetic research purposes does not fall under the Human Research Act (HRA) and therefore does not require ethical approval. Human blood samples were obtained from healthy younger volunteers of both sexes with informed consent and anonymized immediately after donation. Blood was kept at room temperature for up to 8 h or at 4 °C for up to 48 h until production of MT. According to Swiss federal regulations approval of the experimental protocol was not needed. To produce MT, 50 mM CaCl_2_ (Merck, CAS Number 10043–52-4) and 1U/ml thrombin (Sigma, 605,157) was added to 1 ml of citrated whole blood (porcine or human) which was left to clot at 37 °C for 1 h. After 1 h, the clot was placed in a petri dish, and washed with 0.9% NaCl solution (NaCl powder, Sigma S9888 dissolved in dH_2_O). The clot was cut up with two scalpel blades into small fragments. The fragments were resuspended with 0.9% NaCl solution and pipetted on top of a wetted 200 µm filter (PluriSelect, Ref. 43-50200-50) which was placed on top of a wetted 40 µm filter (PluriSelect, Ref. 43-57040-50) on a 50 ml falcon tube. The thrombus fragments were pressed through the 200 µm filter with the back of a 5 ml syringe plunger and washed with 0.9% NaCl solution. This created slightly elongated MT fragments with about 200 µm by 200 µm side dimensions. All fragments on top of the 40 µm filter were washed and resuspended in 0.9% NaCl solution. These MT were used for experiments after 24 h or maximum 48 h storage in 0.9% NaCl solution at 4 °C. For isolation of porcine or human citrate plasma used for perfusion, blood was processed no later than 4 h after withdrawal by centrifugation at 4 °C, 2000G for 15 min and stored at − 20 °C.

### Hematoxylin and Eosin staining of clots

Freshly produced clots (macroscopic) were fixed for 24 h in 10% formaldehyde (Sigma 252549-100ML) and embedded in TissueTek OCT compound (Sakura 4583). 5 µm thin sections were cut and stained with H&E. Tissue sections were imaged on a Leica DMI4000B light microscope at 5 × magnification. Image J software (version 1.54i) was used for quantitative analysis of clot porosity.

### Microthrombus lysis under flow

The in vitro set-up for MT lysis under flow was established with the aim to mimic IC administration of a fibrinolytic drug using a CoFI procedure (CorFlow Therapeutics AG, Baar, Switzerland) as described in Rösch et al.^26^. CoFI is performed using a multi-lumen catheter which occludes the coronary vessel by balloon inflation and infuses medication distally of the balloon occlusion, leading to high local drug concentrations. After at most 90 s, the balloon is deflated allowing drug washout while normal blood flow is resumed (Fig. 1A, top, “CoFI principle”). To mimic this procedure in vitro, MT were incubated within the microfluidic chip for 90 s with different concentrations of alteplase (Actilyse 10 mg, Boehringer-Ingelheim Ref. 303324, diluted in dH2O to 100 µg/ml, 60 µg/ml, 40 µg/ml, 20 µg/ml, 5 µg/ml) or NaCl for controls. After 90 s without flow, the chip was continuously perfused with autologous porcine or pooled human citrate plasma at 37 °C for 25 min (Fig. 1A, bottom, “CoFI in vitro”). Additionally, we tested whether an IV alteplase bolus as performed in the clinical setting^59^, significantly increases thrombolysis compared to CoFI. The IV bolus was mimicked in vitro by perfusion of MT with plasma containing 2 µg/ml alteplase (Fig. 1B, “IV dose in vitro”). To facilitate comparison of CoFI to an IV dose, alteplase concentrations (µg/ml) are converted into alteplase microdoses (mg) as shown in Fig. 1C. Finally, we also included a combined approach (CoFI + IV dose) consisting of 90 s alteplase incubation and perfusion with plasma containing 2 µg/ml alteplase.

For observation of thrombolysis, the microfluidic chip was mounted into a titanium backplate^26^ with channels for infusion and drainage of the microfluidic chip as well as a window for optical access of a backlight (LED Microscopy backlight, 50 mm, 9W, BoliOptics, California, USA). Once mounted, plasma perfusion was achieved using a syringe pump (Harvard Instruments Cat. 70-2212) set to 37 µl/min. After 25 min of perfusion, the syringe pump was stopped, independently of the amount of MT lysis. The flow rate used for the in vitro experiments was calculated using Poiseuille’s law adapted for parallel plate flow, resulting in the following formula:

$\tau =\mu 8Q/(H^2 B)$ where shear stress = τ, height of the channel = H, width of the channel = B, plasma viscosity = µ and flow = Q. Our aim was to achieve a shear stress of 30 dyne/cm^2^, which represents an average physiological shear stress for small arterial vessels as reported in literature^60–62^. Based on the approximate channel geometry, desired shear stress and Poiseuille’s law, the flow rate Q = 37 µl/min was calculated. Additional simulations using COMSOL were performed to verify the shear stress profile more precisely based on the exact channel geometry. The simulations showed that in fact a mean shear stress of 50 dyne/cm^2^ was achieved on average near the microthrombus with a flow rate of 37 µl/min. Based on the calculations and simulations it was concluded that the shear stress in the microfluidic model was within the physiological range for small arterial vessels.

### Microthrombus lysis quantification

Images for lysis quantification were taken during the MT lysis experiments every 5 s using a highspeed camera (Photonfocus, D4096 GigE camera series, resolution 4096 × 3072) and telescopic lense (F 2.8/100 mm Macro Lens, Samyang and intermediate Ring set, Walimex). Quantification of MT lysis from these images was based on the method described in Bizjak et al.^33^. Using Fiji imaging software (version 1.54i), an automatic threshold was applied. The area of the MT was selected manually and the amount of pixels, representing the MT, was measured. Based on this, the percentage decrease in pixels between the last and first image was calculated. To follow MT lysis over time, the same threshold was applied to a stack of images representing one 25-min lysis experiment, and a region of interest (ROI) drawn around the MT. All pixels representing the MT within that ROI were measured over time and percentage decrease was calculated with respect to the first image.

### Influence of different microdoses of alteplase on porcine and human endothelial cells

Primary PAECs or primary HAECs isolated from the thoracic aorta of two different donors were used exclusively until passage four to avoid phenotypic drift. PAECs and HAECs were obtained from a research group at the University of Bern where the cells were isolated according to a previously published protocol^63^. PAECs and HAECs were incubated in vitro with different doses of alteplase used in the perfusion experiment described above to test whether the cells could tolerate elevated local concentrations. For this, primary PAECs or HAECs were grown in chamber slides (Semadeni Ref. 7647) with cell culture medium (DMEM Glutamax, Gibco 21885-025 for PAEC and Large vessel endothelial cell basal medium, Gibco M200500 for HAEC) with 10% heat inactivated fetal bovine serum (FBS, Sigma F7542) and 1% Penicillin/streptomycin (P/S, Gibco 15140-122) until confluence. Cells were then incubated with alteplase (100 µg/ml, 60 µg/ml, 40 µg/ml, 20 µg/ml, 5 µg/ml diluted in PBS or large vessel EC medium, controls without alteplase) for 10 min at 37 °C in a humidified incubator. Subsequently, cells were fixed with a 4% Formaldehyde solution (Sigma 252549-500ML stock solution 37%) and blocked with PBS-3% BSA (bovine serum albumin, Sigma A7030-100G) for 1 h at room temperature. Cells were stained for CD31 (R&D MAB33871, 1:100 dilution) or VE-Cadherin (R&D Systems AF938, 1:100 dilution) and F-Actin AF670 (Cytoskeleton Inc. Ref. PHDN1, 1:200 dilution) diluted in PBS-1%BSA-0.05% Tween 20 (Tween 20, AppliChem A4974,0250) and incubated over night at 4 °C. Secondary antibody against CD31 (Invitrogen, Ref. A-11006, 1:500 dilution) or VE-Cadherin (Invitrogen Ref. A-11001, 1:500 dilution) was diluted in PBS-1%BSA-0.05% Tween 20 and incubated for 1.5 h at room temperature and nuclei were counterstained with DAPI (Sigma, 32670-20MG-F). Slides were mounted using mounting medium (Invitrogen P36934) and visualized with a confocal microscope (Zeiss LSM980).

### Statistical analysis

All graphs were created with GraphPad Prism (version 10). Normality of data was assessed using the Shapiro–Wilk test. Data are represented as mean ± standard deviation. One-way or two-way ANOVA as well as Student’s t-test or Kruskal–Wallis test and Spearman correlation index were used for statistical analysis. Blood from at least five different porcine or human donors was used, and independent experiments were performed at least six times. A difference of *p* < 0.05 was considered significant.

## Electronic supplementary material

Below is the link to the electronic supplementary material.

Supplementary Material 1

## Data Availability

All data are available in the manuscript and supplementary material.
