# Supplementary material for: Dissolving porcine and human microthrombi by short exposure to microdoses of alteplase in an in vitro model of microvascular obstruction
Source: Sci Rep. 2025 May 24;15:18114. doi: 10.1038/s41598-025-03060-1 (PMC12103500; doi:10.1038/s41598-025-03060-1)
Supplement: Supplementary file 1 — Supplementary Material 1 [file 41598_2025_3060_MOESM1_ESM.pdf]

Supplementary Material

Dissolving porcine and human microthrombi by short exposure to microdoses of alteplase in an in vitro model of microvascular obstruction

Anastasia Milusev[1], PhD; Yannick Rösch[1], PhD; Yves Kuster[2]; Petra Wolint[3]; Jens Ulmer[2], PhD; Miriam Weisskopf[4], DVM; Nikola Cesarovic[2, 5], PhD; Dominik Obrist[1], PhD

[1] ARTORG Center for Biomedical Engineering Research, University of Bern, Switzerland

[2] Institute for Microtechnology and Photonics, OST University of Applied Sciences, Buchs SG, Switzerland

[3] Department of Health Sciences and Technology, Swiss Federal Institute of Technology, Zurich, Switzerland

[4] Center for Surgical Research, University Hospital Zurich, University of Zurich, Switzerland

[5] Department of Cardiothoracic and Vascular Surgery, German Heart Center Berlin, Berlin, Germany

A

Alteplase microdose	Slope of clot dissolution		
	CoFI	CoFI + IV alteplase	p-value
Control	0.0023 ± 0.00027	0.0035 ± 0.00037	<0.0001
0.3 mg	0.013 ± 0.00085	0.02 ± 0.00062	<0.0001
0.6 mg	0.014 ± 0.00056	0.019 ± 0.00048	0.85
0.9 mg	0.021 ± 0.00070	0.028 ± 0.00051	0.0162
1.5 mg	0.034 ± 0.00075	0.046 ± 0.00083	<0.0001

B

Alteplase microdose	Slope of clot dissolution		
	CoFI	CoFI + IV alteplase	p-value
Control	0.013 ± 0.00048	0.052 ± 0.000955	<0.0001
0.075 mg	0.03 ± 0.00095	0.04 ± 0.00085	<0.0001
0.3 mg	0.032 ± 0.00102	0.04 ± 0.00095	0.0001
0.6 mg	0.03 ± 0.00074	0.044 ± 0.00111	0.0203
1.5 mg	0.035 ± 0.0013	0.041 ± 0.0012	<0.0001

Supplementary Table S1: Slope of thrombolysis for porcine and human microthrombi over time. Slope of thrombolysis was calculated from lysis over time curves for **(A)** porcine microthrombi and **(B)** human microthrombi incubated with four different microdoses of alteplase or without alteplase (Control). Slopes for controlled flow infusion (CoFI) are compared to the combined approach (CoFI + IV dose). Values for slope steepness and standard deviation are shown. Data are from six to eight independent experiments and five to six different blood donors. Unpaired Student's t-test was used for statistical analysis and $p < 0.05$ was considered significant.

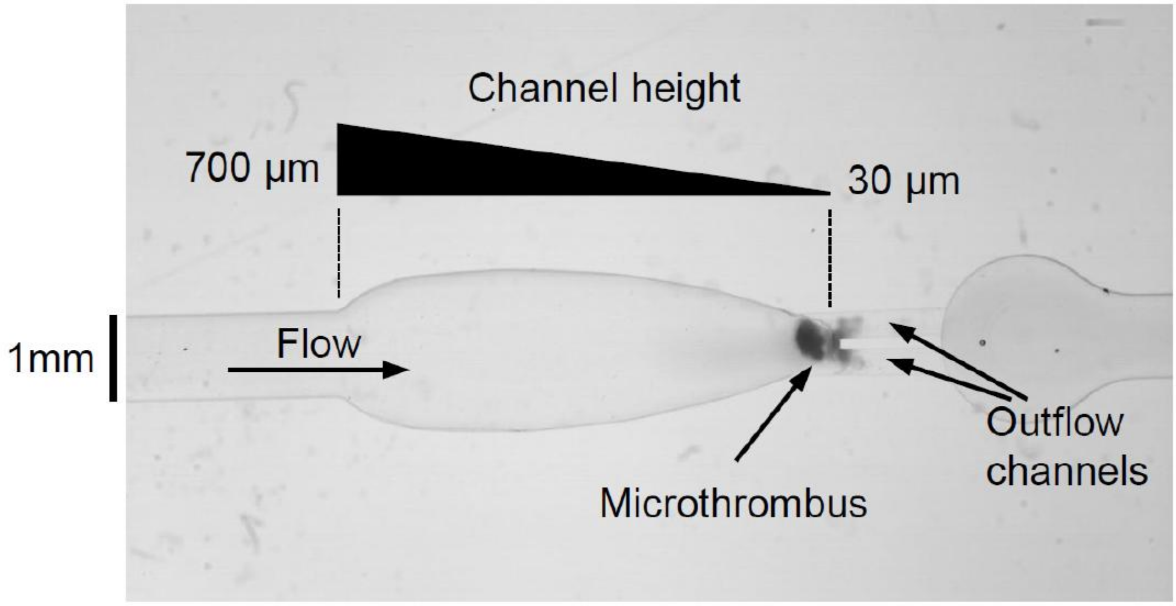

Supplementary Figure S1: Microfluidic chip design to model microvascular obstruction in vitro. Image of the microfluidic chip with a chamber of tapered height (channel height 700 µm – 30 µm) in which a microthrombus is trapped. Two outflow channels (300 µm wide and 30 µm high) assure continuous perfusion without complete channel occlusion after MT injection. Upon perfusion, flow occurs from left to right and is maintained at a constant flow rate of 37 µl/min using a syringe pump.

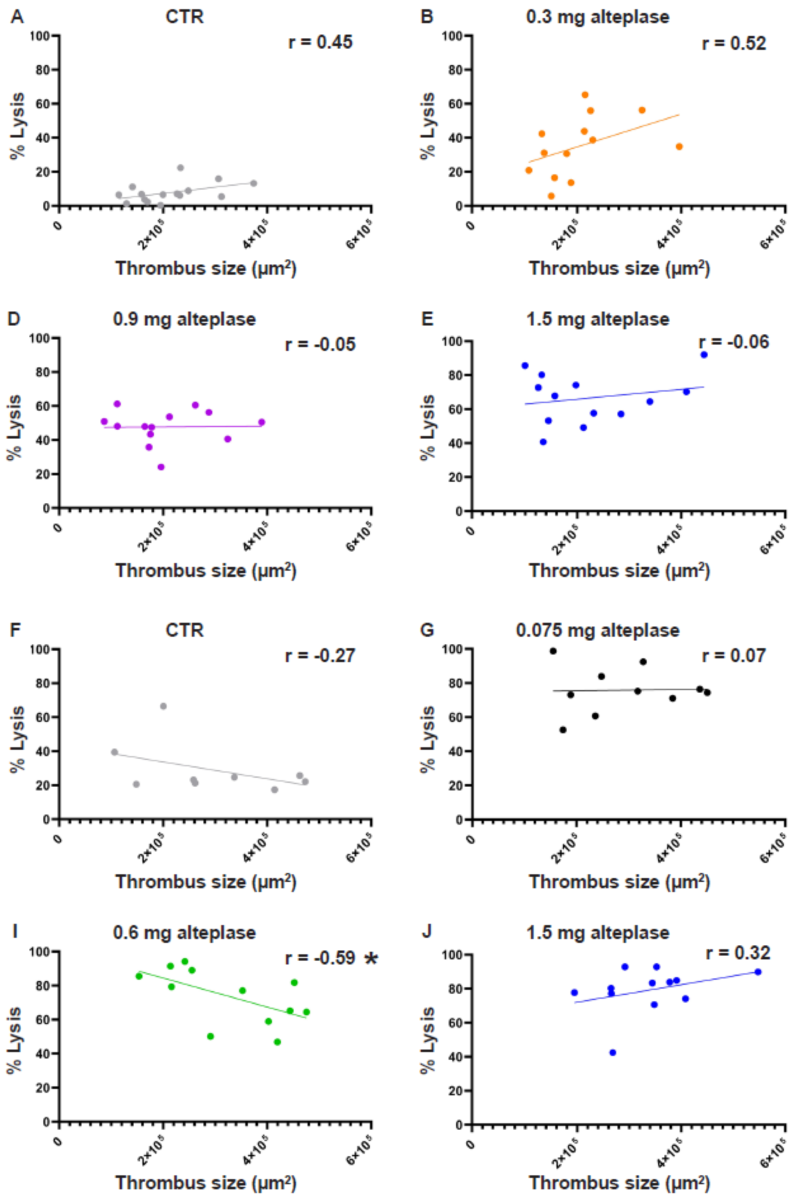

Supplementary Figure S2: Relationship between initial thrombus size and microthrombus lysis. (A-E) porcine MT and **(F-J)** human MT are plotted according to the MT size in µm^{2,} and the corresponding MT lysis achieved at the end of lysis experiments. Linear regression analysis and nonparametric correlation test (Spearman r) was used to determine whether initial MT size had an influence on the amount of lysis for all tested alteplase microdoses as well as controls (not treated with alteplase). $p<0.05$ was considered significant.

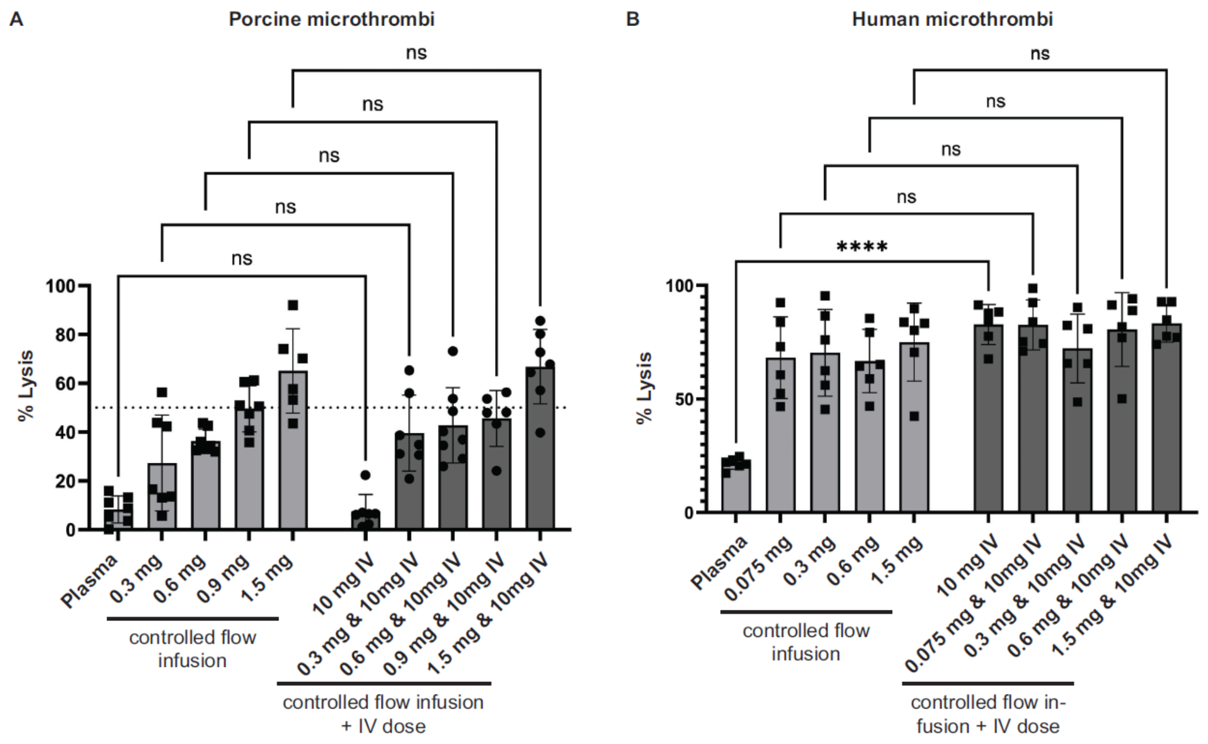

Supplementary Figure S3: MT lysis comparing CoFI to a combined approach. MT lysis after 25 minutes perfusion with citrate plasma (light grey bars) or citrate plasma containing 2 µg/ml alteplase (dark grey bars) for porcine **(A)** or human **(B)** MT. Two-way ANOVA with multiple comparisons was used for statistical analysis. Data are from six to eight independent experiments and five to six different blood donors. **** $p < 0.001$

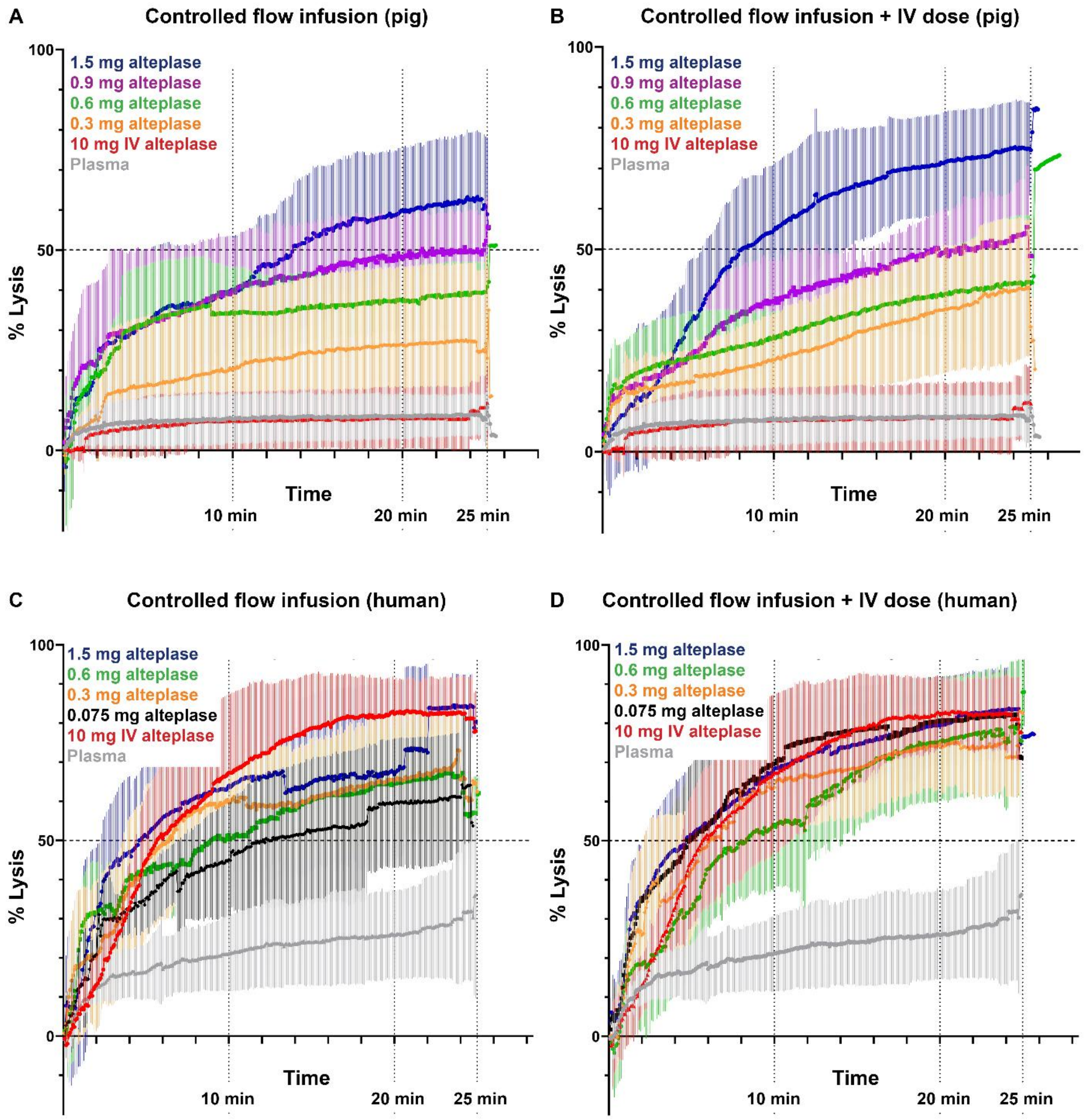

Supplementary Figure S4: Standard deviation of thrombolysis of MT over time comparing controlled flow infusion to a combined approach (CoFI + IV dose). (B, C) Thrombolysis over time for **(A, B)** porcine or **(C, D)** human MT treated with different alteplase microdoses and perfused with **(A, C)** plasma or **(B, D)** plasma containing 2 µg/ml alteplase. Each curve represents the mean lysis over time of at least 6 independent experiments with the corresponding standard deviation.
